# Correction: Harris et al. Derivation of the Omega-3 Index from EPA and DHA Analysis of Dried Blood Spots from Dogs and Cats. *Vet. Sci.* 2023, *10*, 13

**DOI:** 10.3390/vetsci10070422

**Published:** 2023-06-29

**Authors:** William S. Harris, Kristina H. Jackson, Heather Carlson, Nils Hoem, Tonje E. Dominguez, Lena Burri

**Affiliations:** 1 Fatty Acid Research Institute, Sioux Falls, SD 57106, USA; 2 Department of Internal Medicine, Sanford School of Medicine, University of South Dakota, Sioux Falls, SD 57105, USA; 3 OmegaQuant Analytics, LLC., Sioux Falls, SD 57106, USA; 4 All-City Pet Care Veterinary Emergency Hospital, Sioux Falls, SD 57105, USA; 5 Aker BioMarine Antarctic AS, NO-1327 Lysaker, Norway

## Error in Table

In the original publication [1], there was a mistake in Table 1 as published. All four values for dogs shown in the table are incorrect. The corrected Table 1 appears below.

**Table 1:** Equations to convert DBS EPA + DHA (x) into the Omega-3 Index (RBC EPA + DHA %) (y) by species.

Species	Equation	R^2^	O3I Equivalent of a DBS EPA + DHA of 4%	Approximate Range of O3I Values
Dog (*n* = 33)	y = 0.709x + 0.0002	0.98	2.9%	0.3 to 7%
Cat (*n* = 10)	y = 0.690x + 0.0014	0.96	2.9%	0.5 to 4%
Human (*n* = 100)	y = 1.114x + 0.014	0.88	5.9%	2 to 12%

## Error in Figure

In the original publication [1], there was a mistake in Figure 1. The published graph mistakenly shows the relationship between DBS EPA + DHA and plasma EPA + DHA (which is correctly shown in Supplementary Figure S2). The corrected Figure 1 shows the relationship between DBS EPA + DHA and RBC EPA + DHA as stated in the Figure legend.

## Text Correction

There was an error in the original publication [1]. The third sentence in the second paragraph of the Discussion incorrectly states, “For example, a DBS EPA + DHA value of 4% would translate into an O3I of 5.9% for humans, but 2.4% for dogs and 2.9% for cats”.

The following correction has been made: “For example, a DBS EPA + DHA value of 4% would translate into an O3I of 5.9% for humans, but 2.9% for dogs and cats”.

The authors apologize for any inconvenience caused and state that the scientific conclusions are unaffected. This correction was approved by the Academic Editor. The original publication has also been updated.

## Figures and Tables

**Figure 1:**
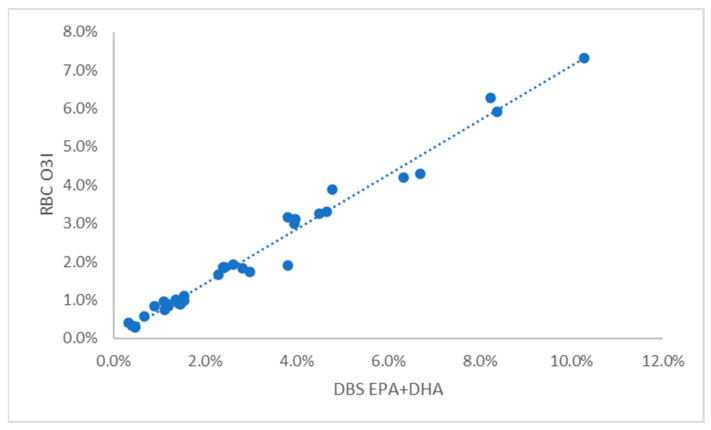
Relationship between the EPA + DHA content of DBS samples and the Omega-3 Index in 33 dogs. DBS, dried blood spot; RBC, red blood cell.
