# Supplementary material for: Derivation of the Omega-3 Index from EPA and DHA Analysis of Dried Blood Spots from Dogs and Cats
Source: Vet Sci. 2022 Dec 26;10(1):13. doi: 10.3390/vetsci10010013 (PMC9863621; doi:10.3390/vetsci10010013)
Supplement: Supplementary file 1 [file vetsci-10-00013-s001.zip › vetsci-2101929-supplementary.pdf]

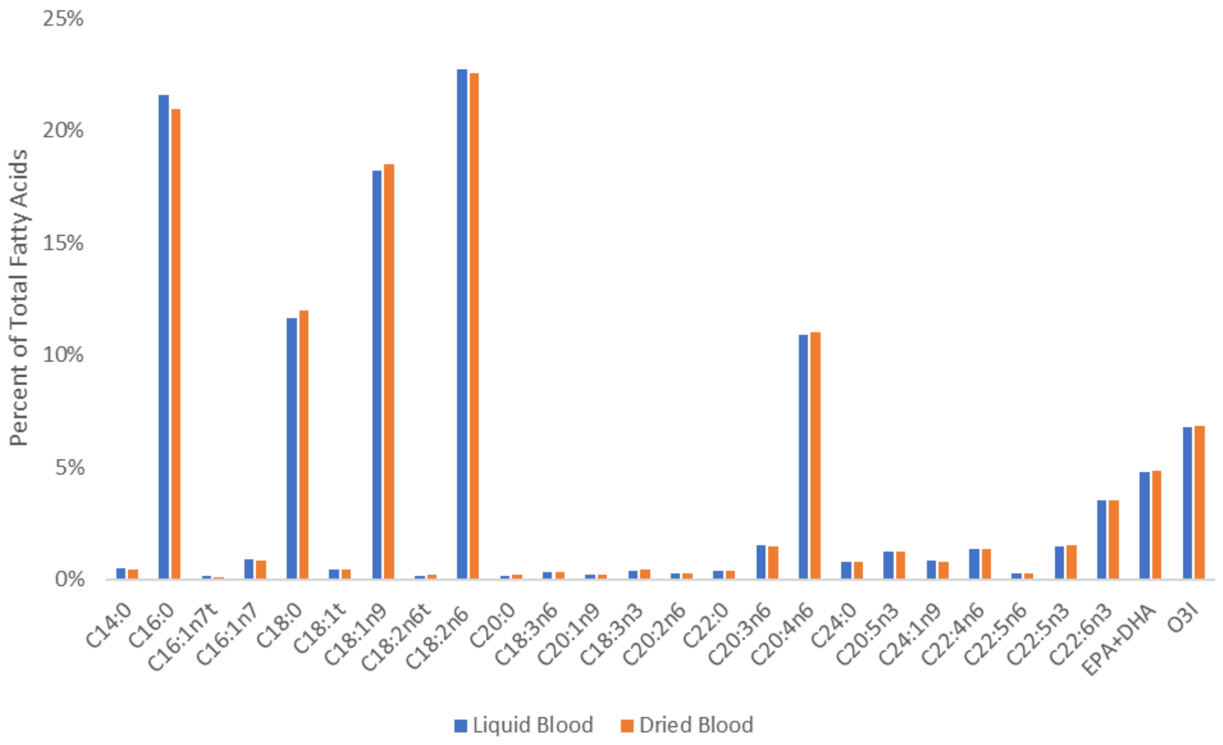

Figure S1. Mean fatty acid composition of liquid vs dried blood spot analysis from human samples.

These data were obtained by randomly choosing 30 whole, liquid blood samples received from patients at OmegaQuant Analytics. A 20 uL sample of whole blood was transferred to a test tube, and then a 50 uL sample was placed on a blood spot card and allowed to dry. Both samples were then analyzed for fatty acid composition in the same run by the same laboratory method as described in the main paper.

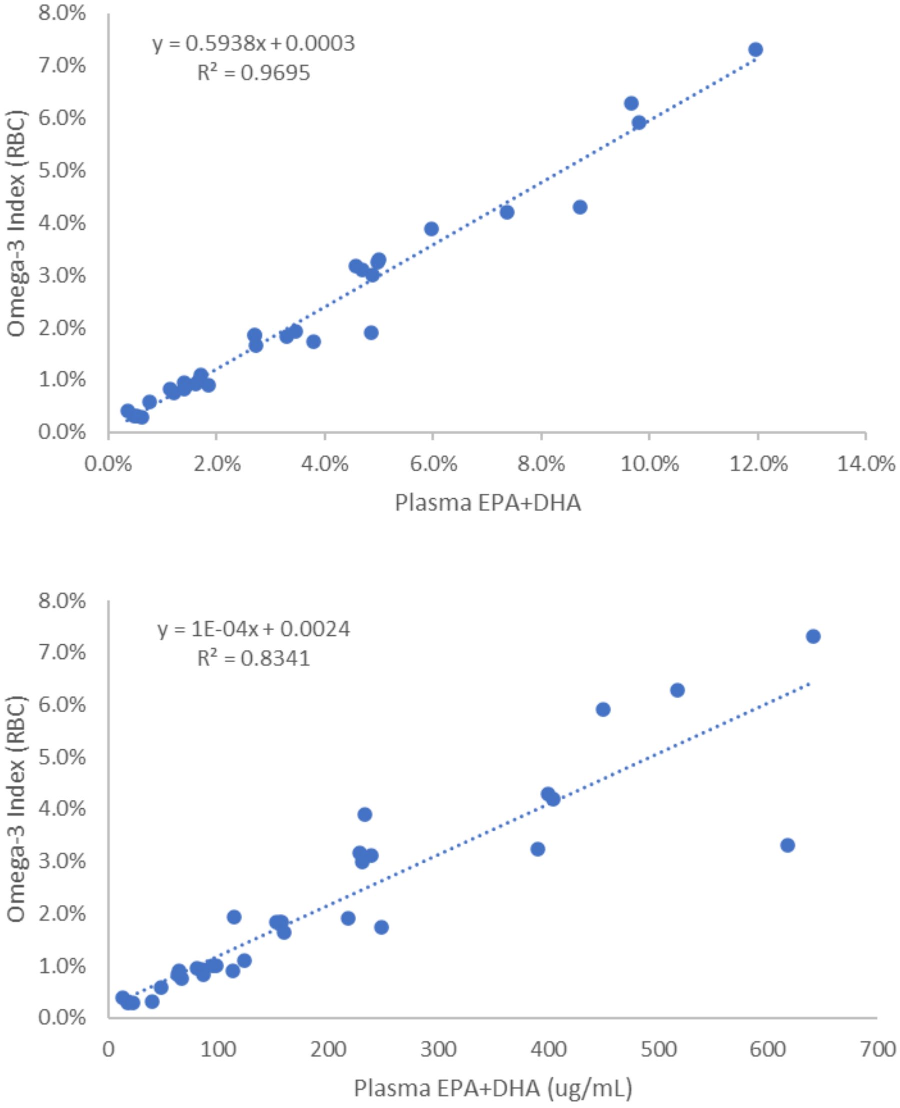

Figure S2. Relations between the Omega-3 Index and plasma % EPA+DHA (top) and plasma EPA+DHA (ug/mL, bottom) in Dogs (n=33)

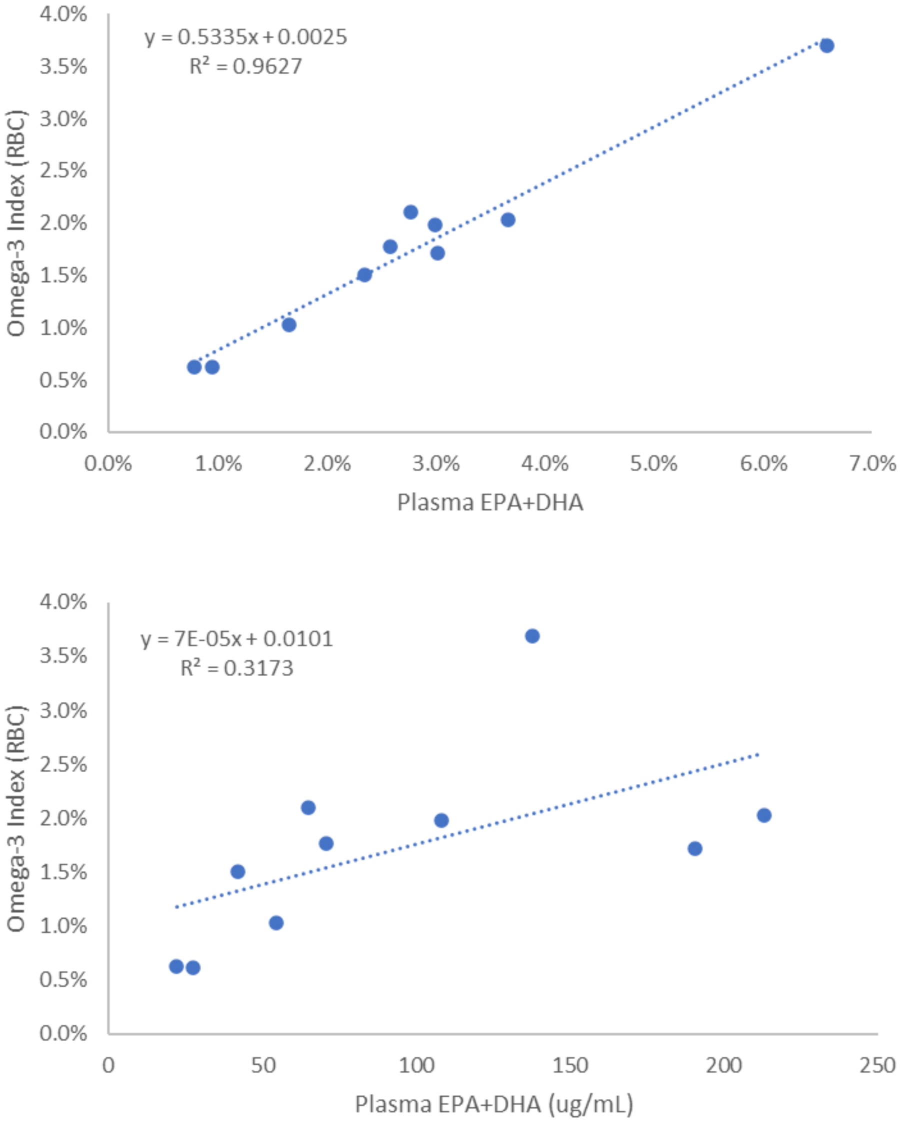

Figure S3. Relations between the Omega-3 Index and plasma % EPA+DHA (top) and plasma EPA+DHA (ug/mL, bottom) in Cats (n=10)

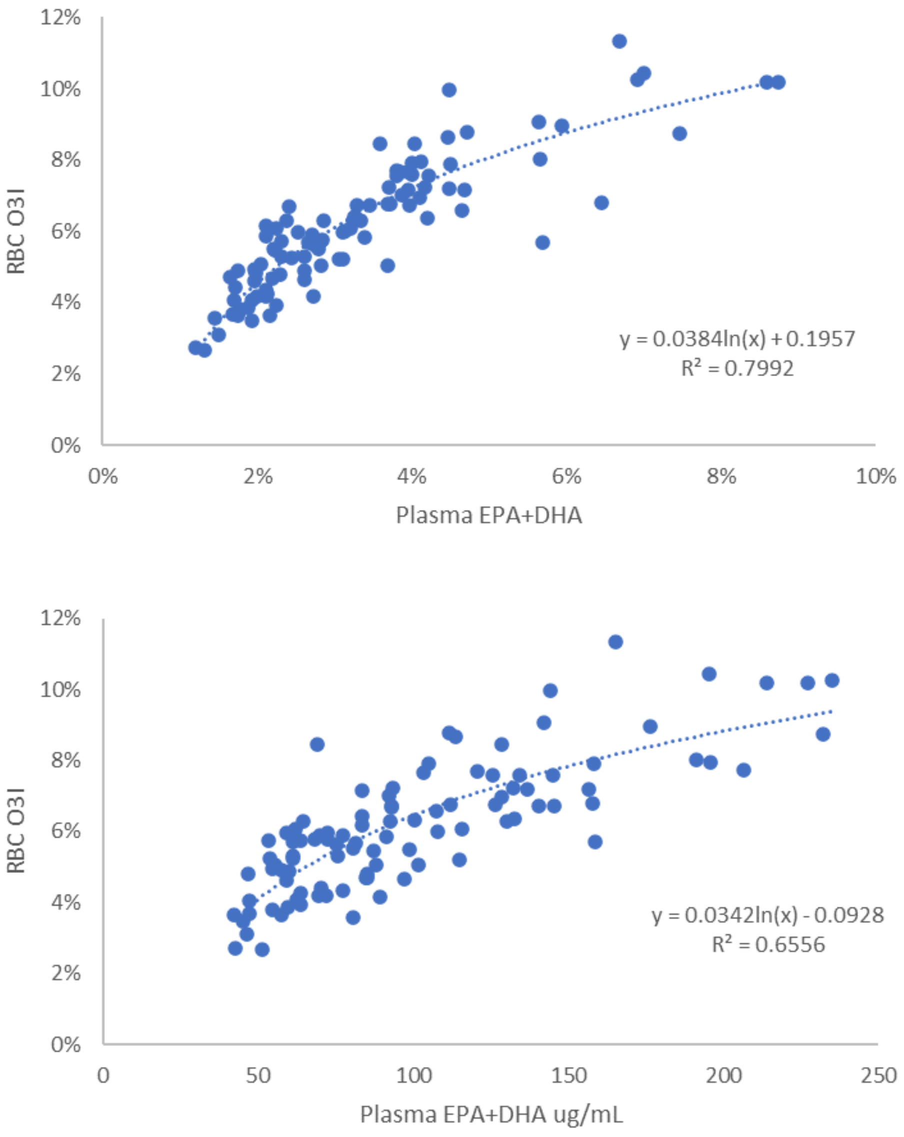

Figure S4. Relations between RBC EPA+DHA (RBC %) and plasma EPA+DHA (%) (top) and plasma EPA+DHA (ug/mL; bottom). Data from 100 random human blood samples.
